# Diagnostic efficacy of [^18^F]FDG PET/CT and [^18^F]FDG PET/MRI in preoperative staging of locoregional urinary bladder cancer: a systematic review and Meta-Analysis

**DOI:** 10.1007/s12672-025-03020-1

**Published:** 2025-07-01

**Authors:** Ahmed Saad Abdlkadir, Dhuha Al-Adhami, Sudqi Allouzi, Mohannad Badarneh, Mohammed Shahait, Marwa Abdulrahman, Saad Ruzzeh, Serin Moghrabi, Akram Al-Ibraheem

**Affiliations:** 1 Department of Nuclear Medicine, King Hussein Cancer Center (KHCC), Queen Rania Street, Al Jubeiha, Amman, 11941 Jordan; 2 Department of Surgery, Clemenceau Medical Center, Dubai, UAE; 3 School of Medicine, The University of Jordan, Amman, 11942 Jordan

**Keywords:** [^18^F]FDG, PET/MRI, PET/CT, Bladder cancer, Urothelial cancer, Meta-analysis

## Abstract

**Background:**

This systematic review and meta-analysis aims to evaluate the diagnostic utility of [^18^F]Fluorodeoxyglucose ([^18^F]FDG) PET/CT and [^18^F]FDG PET/MRI in preoperative locoregional staging of bladder cancer (BC).

**Methods:**

A comprehensive search of PubMed and Scopus databases was conducted up to February 26, 2025, to collect and analyze diagnostic accuracy parameters. Statistical analyses, including publication bias assessment, interstudy heterogeneity evaluation, and effect size adjustment, were performed using Stata software, with a significance threshold set at *p* < 0.05.

**Results:**

A total of 25 eligible studies were identified. Among these, three studies utilized [^18^F]FDG PET/MRI for primary tumor detection, yielding a pooled sensitivity of 90%. In contrast, five studies employing [^18^F]FDG PET/CT for the same purpose reported a pooled sensitivity of 69%. Notably, [^18^F]FDG PET/MRI demonstrated threefold greater effectiveness in primary tumor detection compared to [^18^F]FDG PET/CT based on results of indirect comparison of true positive odds ratio between the two modalities. For lymph node staging, 18 studies employed [^18^F]FDG PET/CT, with pooled sensitivity and specificity estimates of 50% and 91%, respectively. Significant interstudy heterogeneity was observed in lymph node staging, primarily attributed to variations in PET/CT protocols. Studies incorporating bladder priming strategies, dual-time-point imaging, and contrast-enhanced CT protocols achieved superior sensitivity and specificity compared to traditional PET/CT approaches.

**Conclusion:**

The findings highlight the compelling potential of [^18^F]FDG PET/MRI for primary tumor staging in BC. Further research is warranted to explore this innovative modality’s role in locoregional BC evaluation. Additionally, substantial heterogeneity in [^18^F]FDG PET/CT protocols underscores the need for standardization to improve sensitivity. Thus, Future studies should focus on addressing these limitations by adopting optimized PET/CT protocols.

**Supplementary Information:**

The online version contains supplementary material available at 10.1007/s12672-025-03020-1.

## Introduction

Urinary bladder carcinoma (BC) poses a significant health challenge across different geographical regions. In developed nations, it is the second most common urological malignancy after prostate cancer [1]. On a global scale, BC has risen from the tenth to the ninth most commonly diagnosed cancer, with a concomitant increase in both incidence and mortality rates [2, 3]. In 2022, an estimated 614,298 new cases were diagnosed worldwide, representing a 7.1% increase compared with 2020 [2]. The incidence and mortality rates are approximately three to four times higher in men than in women globally [2].

Approximately 80% of BC cases are classified as non-muscle invasive BC (NMIBC), while the remaining 20% are muscle invasive BC (MIBC) [4]. Despite the favorable prognosis associated with NMIBC, over 50% of patients experience disease recurrence following initial treatment, and up to 30% progress to MIBC. While NMIBC generally has a favorable prognosis with appropriate management, MIBC is associated with poorer outcomes, particularly in patients with lymph node or distant metastasis [5]. Therefore, timely diagnosis and accurate staging are crucial for guiding treatment strategies and optimizing patient outcomes.

[^18^F]Fluorodeoxyglucose ([^18^F]FDG) positron emission tomography/computed tomography (PET/CT) has been investigated as a functional imaging modality for tumor staging in various cancers [6]. However, its utility in BC staging is limited by high urinary excretion activity in the bladder and ureters and is further limited by suboptimal sensitivity for locoregional disease evaluation [7]. Despite these limitations, it has emerged as an effective tool to evaluate the whole body and exclude the possibility of distant metastasis, proving effective in challenging scenarios of multiple neoplasia and improving overall management intent when provided with a multimodal imaging approach [8–10]. Consequently, current guidelines put forth by the American College of Radiology endorse the utilization of [^18^F]FDG PET/CT for BC staging while acknowledging the radiation risk and aforementioned limitations associated with this modality [11].

On the other hand, [^18^F]FDG PET/MRI represents an innovative molecular imaging approach that promises improved lesion delineation and lower radiation exposure and may mitigate some of the limitations encountered with [^18^F]FDG PET/CT [12]. While its availability is currently limited to a few nuclear medicine institutions worldwide, literature-based evidence and meta-analyses on various cancer subtypes in challenging anatomical locations, such as the head and neck and pelvis, have demonstrated the great potential of this novel hybrid imaging approach [13, 14]. To date, there is no collective evidence to support the use of this novel approach, as a comprehensive analysis of studies addressing its potential for locoregional BC evaluation has not been performed [11].

This systematic review and meta-analysis aims to serve multiple purposes. First, it aims to update our current knowledge on the diagnostic accuracy of [^18^F]FDG PET/CT in the locoregional staging of BC. Second, this study provides the first meta-analysis of the diagnostic efficacy of PET/MRI in BC locoregional disease staging and provides an indirect comparison between the two modalities to assess whether any significant difference exists between them.

## Methods

### Data sources and search strategy

A systematic literature search of the PubMed and Scopus databases was independently performed by three authors (ASA, DA-A, and AA-I) to identify studies assessing the diagnostic accuracy of hybrid imaging, with [^18^F]FDG PET as the primary radiotracer, in the staging of BC. A comprehensive search algorithm was implemented, incorporating various MeSH key terms (Supplementary Table 1) [15]. The final search update was conducted on February 26, 2025. The inclusion criteria were limited to original articles specifically addressing the topic of interest in BC patients within clinical settings prior to surgery. During the initial screening phase, duplicate studies, review articles, book chapters, conference papers, abstracts, preclinical studies, and irrelevant articles were excluded. The full texts of potentially relevant studies were retrieved for detailed evaluation. Data extracted from the studies were imported into Microsoft Excel Professional Plus 2024 software (Redmond, Washington, United States) for organization, sorting, screening, and filtering. This systematic review and meta-analysis was conducted in accordance with the Preferred Reporting Items for Systematic Review and Meta-Analysis Protocols (PRISMA-P) guidelines and was registered in the International Prospective Register of Systematic Reviews (PROSPERO; registration number CRD420251006969) [16]. In its investigation of diagnostic test accuracy metrics, this systematic review and meta-analysis conform to the current standards outlined in the most recent version of the Updated List of Essential Items for Reporting Diagnostic Accuracy Studies (STARD) protocol (Supplementary Tables 2–3). Data collection and management were conducted by three authors (ASA, DAA, and AAI). Any disagreements among the authors were addressed through discussion until a consensus was achieved.

### Data collection

A comprehensive retrieval and analysis of studies meeting the predefined inclusion criteria for this systematic review and meta-analysis was performed. A dedicated Microsoft Excel spreadsheet was developed to facilitate a systematic and in-depth examination of the selected articles. Key data were extracted from each study, including the first author’s name; publication year; corresponding author’s address; study design; patient cohort size; median age; prior lines of therapy; [^18^F]FDG PET modality; [^18^F]FDG PET imaging time; injected dose of [^18^F]FDG; a comprehensive description of the [^18^F]FDG PET protocol; reference standard used for comparison; and numerical data necessary for the calculation of sensitivity, specificity, and accuracy.

### Assessment of methodological quality

Three authors (ASA, DA-A, and AA-I) utilized the Quality Assessment of Diagnostic Accuracy Studies 2 (QUADAS-2) criteria to assess the methodological quality of the included studies. The QUADAS-2 tool was used to evaluate the risk of bias and applicability concerns across the patient selection, index test, and reference standard domains. Specifically, the flow and timing domain was used to evaluate the risk of bias [17].

### Statistical analysis

Pooled estimates of diagnostic performance (Supplementary Table 2), including sensitivity, specificity, and the diagnostic odds ratio (DOR), are presented as point estimates with corresponding 95% confidence intervals (CIs). An indirect comparative analysis using Bucher’s method was performed to assess the statistical difference in the diagnostic efficacy of [¹⁸F]FDG PET/CT versus [¹⁸F]FDG PET/MRI based on the difference in true positive odds ratios of the two modalities (Supplementary Table 3) [18]. Between-study heterogeneity was statistically assessed via the inconsistency index (I^2^). An I^2^ value less than 50% was interpreted as indicative of low to moderate heterogeneity, whereas values exceeding 50% suggested substantial to high heterogeneity [19]. For the analysis and pooling of diagnostic performance measures, a random effects model was employed. This model was utilized to generate summary receiver operating characteristic (SROC) curves and to estimate the area under the curve (AUC). Publication bias was evaluated via Egger’s test. A minimum of ten studies were required to consider visual representation of publication bias via funnel plot analysis [20]. Statistical significance was defined as a *p* value of less than 0.05. In instances of substantial statistical heterogeneity, subgroup meta-analyses were performed to identify potential sources of bias. Pooled estimates were also calculated for subgroups of studies defined according to specific study designs and parameters. All the statistical analyses were performed via Stata software, version 17.0 (College Station, Texas).

## Results

**Fig. 1:**
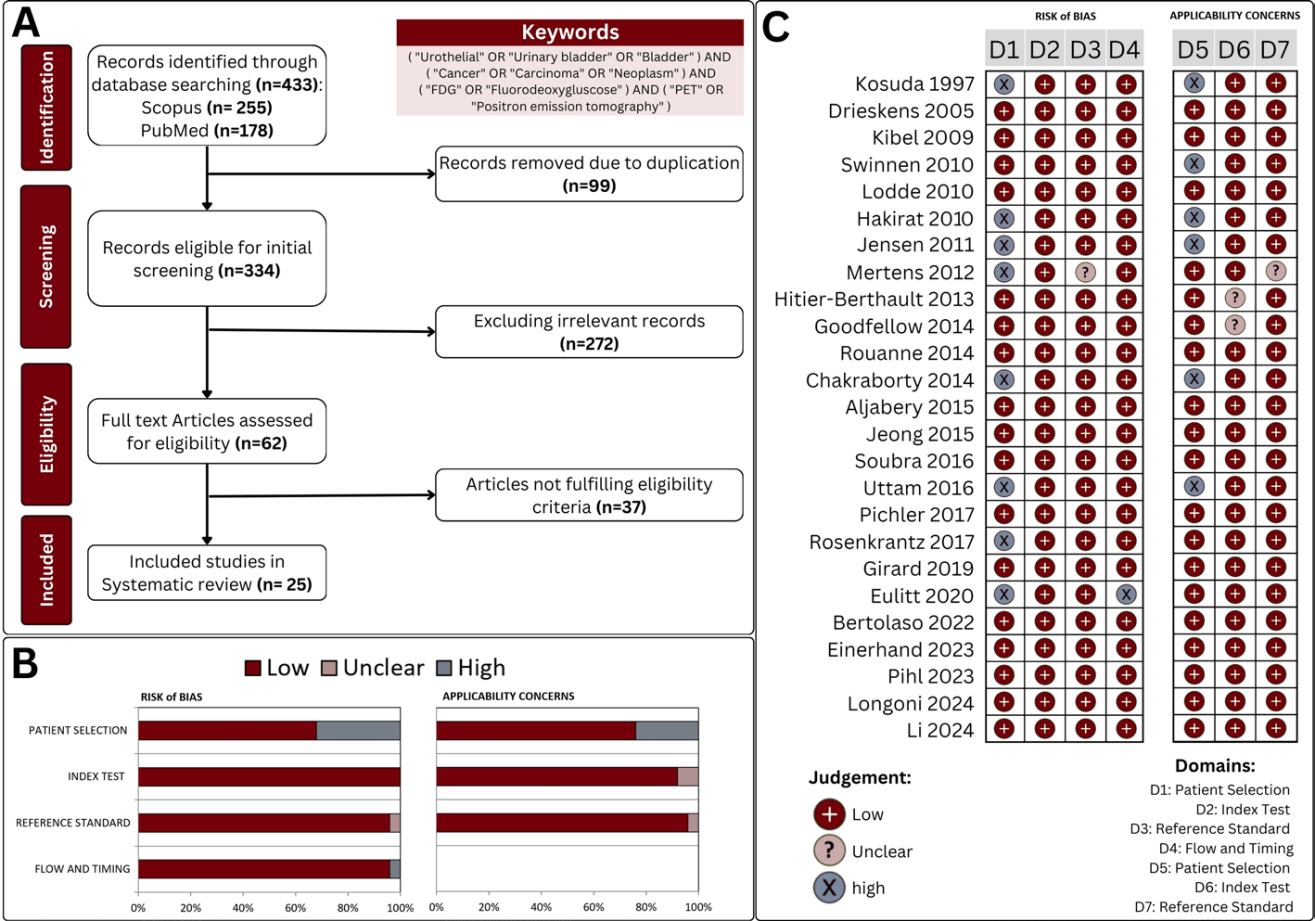
**A** Comprehensive flowchart illustrating the selection criteria utilized for the inclusion of studies in the analysis. **B-C** Findings from the evaluation of bias risk and applicability concerns for the studies included in this analysis, utilizing the QUADAS-2 criteria

The systematic literature search yielded a total of 433 research articles. Following the removal of duplicates (*n* = 99) and the exclusion of 272 articles on the basis of title and abstract screening, 62 potentially relevant articles were retrieved for full-text review. Twenty-five articles were ultimately deemed eligible for inclusion in the systematic review on the basis of predefined inclusion criteria (Fig. 1A) [21–45].

### Methodological quality

The methodological quality of the included studies was assessed via the QUADAS-2 criteria (Fig. 1B, C). Several studies demonstrated a high risk of bias in the patient selection domain [21, 22, 25, 28, 32, 35, 37, 40, 43]. Furthermore, two studies presented unclear applicability concerns related to the index test due to incomplete reporting of the protocol [36, 38]. Finally, one study introduced uncertainty regarding the reference standard, affecting both the risk of bias and applicability domains [28].

### Systematic review

The studies included in this systematic review were published between 1997 and 2024 [21–45]. Sixteen studies were conducted retrospectively [21–23, 26, 27, 29, 31, 32, 35, 37, 38, 41–45], whereas nine were conducted prospectively [24, 25, 28, 30, 33, 34, 36, 39, 40]. The aggregate patient population comprised 1656 individuals, with a male predominance (80%). Geographically, 54% of the studies were conducted in Europe [22, 24, 26–29, 35, 36, 38, 39, 41, 42, 44, 45]. The majority of studies (*n* = 22) employed [^18^F]FDG PET/CT as the primary diagnostic modality, whereas only three studies utilized [^18^F]FDG PET/MRI (Table 1).

**Table 1:** General characteristics of included studies

Study Name	Country	Patients	Study Nature	Index Test	Reference Standard
Kosuda 1997	JP	12 (7 M, 5 F)	OR	PET/CT	HP
Drieskens 2005	BE	55 (47 M, 8 F)	OR	PET/CT	HP or CF
Kibel 2009	US	42 (32 M, 11 F)	OP	PET/CT	HP
Swinnen 2010	BE	51 (43 M, 8 F)	OR	PET/CT	HP
Lodde 2010	CA	70 (57 M, 13 F)	OP	PET/CT	HP or CF
Hakirat 2010	IN	22 (19 M, 3 F)	OR	PET/CT	HP
Jensen 2011	DK	18 (14 M, 4 F)	OR	PET/CT	HP
Mertens 2012	NL	19 (14 M, 5 F)	OP	PET/CT	HP or CT
Hitier-Berthault 2013	FR	52 (44 M, 8 F)	OP	PET/CT	HP
Goodfellow 2014	UK	93 (70 M, 23 F)	OR	PET/CT	HP
Rouanne 2014	FR	102 (80 M, 22 F)	OP	PET/CT	HP
Chakraborty 2014	IN	23 (20 M, 3 F)	OR	PET/CT	HP
Aljabery 2015	SE	54 (47 M, 7 F)	OR	PET/CT	HP
Jeong 2015	KR	61 (46 M, 15 F)	OP	PET/CT	HP
Soubra 2016	US	78 (63 M, 15 F)	OR	PET/CT	HP
Uttam 2016	IN	15 (14 M, 1 F)	OR	PET/CT	HP
Pichler 2017	AT	70 (53 M, 17 F)	OR	PET/CT	HP
Rosenkrantz 2017	US	22 (19 M, 3 F)	OP	PET/MRI	HP
Girard 2019	FR	61 (56 M, 17 F)	OP	PET/CT	HP
Eulitt 2020	US	18 (15 M, 3 F)	OP	PET/MRI	HP
Bertolaso 2022	FR	85 (74 M, 9 F)	OR	PET/CT	HP
Einerhand 2023	NL	237 (174 M, 63 F)	OR	PET/CT	HP
Pihl 2023	SE	157 (116 M, 41 F)	OR	PET/CT	HP
Longoni 2024	IT	199 (168 M, 31 F)	OR	PET/CT	HP
Li 2024	CN	40 (32 M, 8 F)	OR	PET/MRI	HP

AT, Austria; BE, Belgium; CA, Canada; CF, Clinical Follow-up; CN, China; CT, computed tomography; DK, Denmark; F, Female; FR, France; HP, Histopathology; IN, India; IT, Italy; JP, Japan; KR, South Korea; M, Male; NL, Netherlands; OP, Original prospective; OR, Original retrospective; PET/CT, Positron emission tomography/computed tomography; PET/MRI, Positron emission tomography/magnetic resonance imaging; SE, Sweden; UK, United Kingdom; US, United States

## Meta-analysis of primary bladder tumor detection

### Detection rate: [^18^F]FDG PET/CT

**Fig. 2:**
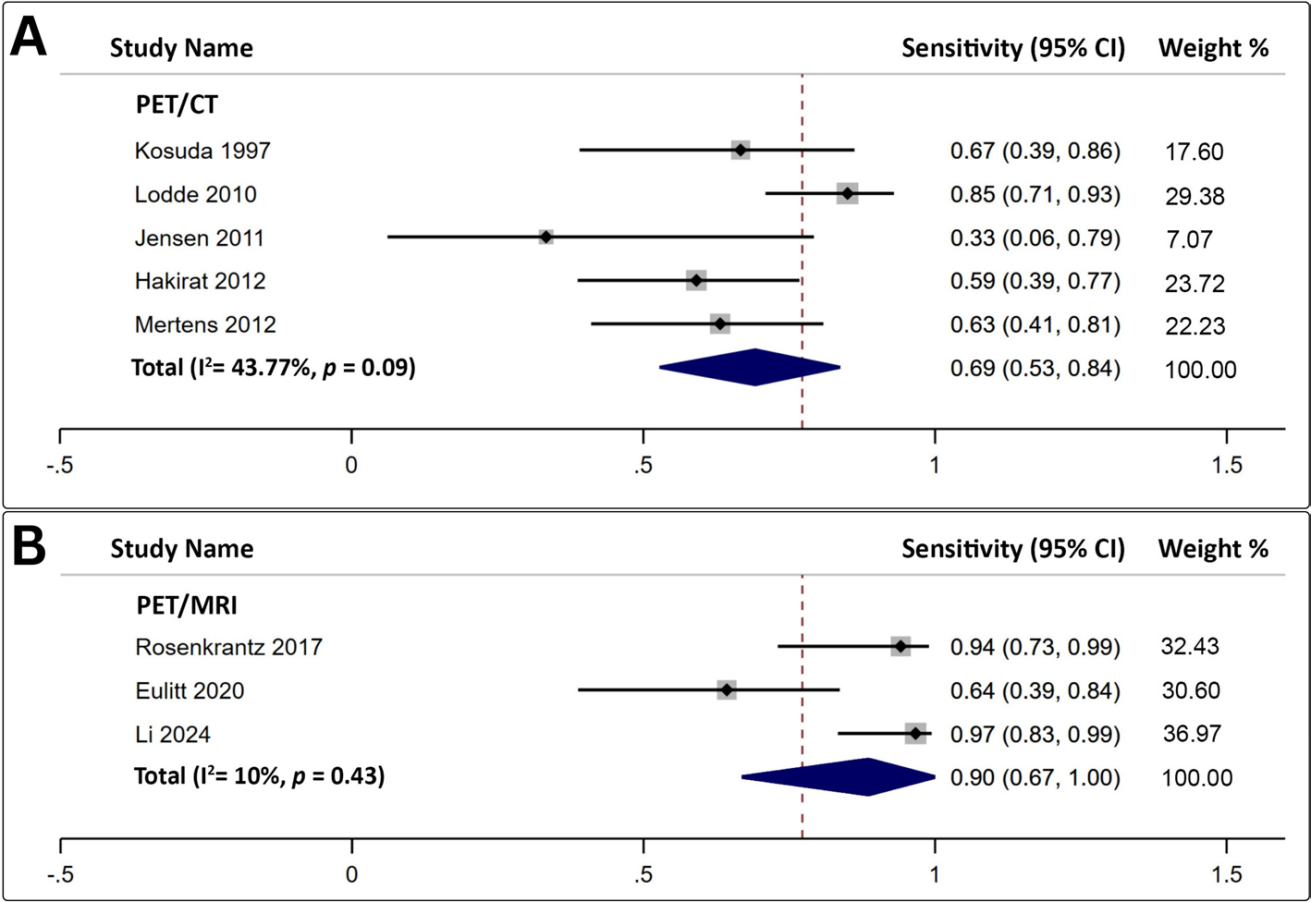
Forest plot analysis of **A** [^18^F]FDG PET/CT, and **B** [^18^F]FDG PET/MRI for the assessment of primary lesions in urinary bladder carcinoma

A total of five studies, encompassing 141 patients, investigated the utility of [^18^F]FDG PET/CT in the evaluation of primary lesion detectability [28, 30, 32, 35, 37]. A per-patient analysis revealed that the overall pooled estimate for intravesical BC lesion detectability was 69% (95% CI: 53–84%). No significant heterogeneity was observed (Fig. 2A). The evaluation of publication bias via Egger’s test was statistically insignificant (*p* = 0.39).

### Detection rate: [^18^F]FDG PET/MRI

Only three studies, including 80 patients, employed [^18^F]FDG PET/MRI for intravesical lesion detection [25, 31, 40]. A per-patient analysis revealed that the overall pooled estimate for intravesical BC lesion detectability was 90% (95% CI: 67–100%). No significant heterogeneity was observed (Fig. 2B). Assessment of publication bias via Egger’s test was statistically insignificant (*p* = 0.61).

### True positive odds ratio: indirect comparison

Overall, the pooled true-positive odds ratio for [^18^F]FDG PET/CT was 0.7 (95% CI: 0.3–1.2). In contrast, [18F]FDG PET/MRI demonstrated a higher true-positive odds ratio of 2.4 (95% CI: 1.4–3.5). Bucher’s indirect comparison revealed statistically superior diagnostic performance for [^18^F]FDG PET/MRI, with an indirect odds ratio of 3.1 (95% CI: 1.5–7.7), indicating a threefold increase in intravesical lesion detection efficacy compared with [^18^F]FDG PET/CT (Table 2).

**Table 2:** Results of indirect odds ratio

Imaging modality	Odds ratio	95% CI	*p*-value
[^18^F]FDG-PET/MRI	2.4	1.4–3.5	0.0001
[^18^F]FDG-PET/CT	0.7	0.3–1.2	
Indirect Comparison	3.1	1.5–7.7	

## Meta-analysis of lymph node staging

### Lymph node staging by [^18^F]FDG PET/CT

**Fig. 3:**
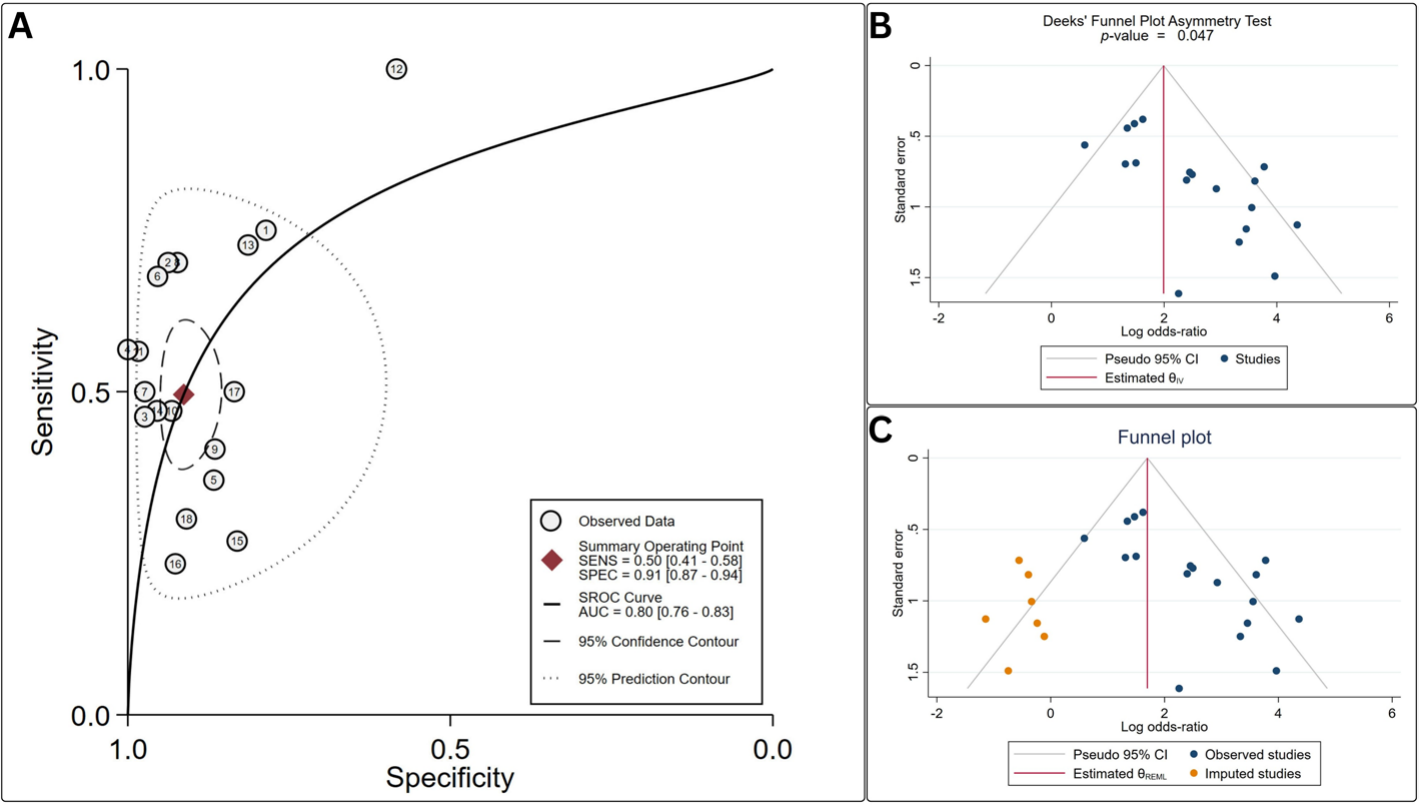
**A** SROC curves for [^18^F]FDG PET/CT in assessing nodal involvement in urinary bladder cancer. **B** Deeks’ funnel plot revealed significant asymmetry in publication trends, indicating a potential inflation of effect sizes. **C** Proposed Deeks’ funnel incorporating adjustments to the effect sizes through the trim-and-fill methodology

A total of 18 studies involving 1,505 BC patients evaluated [18 F]FDG PET/CT for lymph node staging. Per-patient analysis revealed a pooled sensitivity of 50% (95% CI: 41–58%) and specificity of 91% (95% CI: 87–94%). SROC analysis demonstrated an accuracy of 80% (Fig. 3A), with a diagnostic odds ratio (DOR) of 10 (95% CI: 6–18). Substantial heterogeneity (I² > 52%) was observed across all diagnostic accuracy parameters (Table 3). Publication bias assessment revealed significant asymmetry on Deeks’ funnel plot (Egger’s test, *p* = 0.047), suggesting small-study effects favoring inflated effect sizes (Fig. 3B). Trim-and-fill adjustment imputed seven omitted studies with negative outcomes (Fig. 3C), reducing the pooled effect size from 2.3 to 1.7 (Table 4).

**Table 3:** Results of meta-analysis of diagnostic test accuracy for patient-based lymph node staging of bladder cancer patients

Parameter	Pooled Estimate	95% CI	I² (%)	Heterogeneity (*p*)
Sensitivity	50%	41–58%	85%	0.00001
Specificity	91%	87–94%	67%	0.0001
Positive Likelihood Ratio	5.7	3.8–8.7	53%	0.004
Negative Likelihood Ratio	0.6	0.5–0.7	61%	0.0002
Diagnostic Odds Ratio	10	6–18	54%	0.004

**Table 4:** Results of Trim-and-fill analysis. ES, effect size

Meta-analysis	Studies	Missing Studies	ES (95% CI)	Adjusted ES (95% CI)
Diagnosis of nodal disease	18	7	2.31 (1.78–2.83)	1.69 (1.09–2.30)

### Subgroup meta-analysis

Subgroup meta-analyses were performed to identify sources of heterogeneity in [^18^F]FDG PET/CT diagnostic accuracy for lymph node staging. Covariates included age group, sample size, study design, CT protocol, dual-time-point PET/CT use, and bladder emptying strategies. Age group, sample size, and PET protocol variations contributed to subgroup heterogeneity (> 60%). Smaller sample size studies demonstrated significantly higher sensitivity than larger cohorts. Dual-time-point PET/CT protocols yielded superior specificity compared to single-time-point protocols. Enhanced sensitivity trends were observed in studies utilizing contrast-enhanced CT, dual-time-point PET/CT, or bladder emptying strategies; however, these differences lacked statistical significance (*p* > 0.2). Other subgroups showed minimal differences (Table 5).

**Table 5:** Results of subgroup meta-analyses

Covariates	Category	Number of studies	Pooled estimates				I^2^
			Sensitivity % (95% CI)	*p*	Specificity % (95% CI)	*p*	
Age Group	Geriatric	12	49 (39–59)	0.86	93 (90–96)	0.10	61%
	Old age	6	51 (35–67)		84 (76–93)		
Sample Size	> 50	15	46 (38–54)	0.03	92 (89–95)	0.58	67%
	< 50	3	75 (55–96)		86 (72–99)		
Study Design	R	12	49 (39–60)	0.93	89 (85–93)	0.06	47%
	P	6	50 (36–64)		95 (92–99)		
Contrast enhancement	Not applied	12	46 (37–55)	0.22	88 (83–94)	0.41	15%
	Applied	6	59 (44–75)		90 (83–97)		
Delayed imaging	Not applied	15	47 (38 56)	0.18	81 (67–94)	0.04	66%
	Applied	3	62 (40–83)		92 (90–96)		
Bladder Priming	Applied	11	55 (44–65)	0.21	91 (86–96)	0.72	23%
	Not applied	7	42(31–53)		92 (87–96)		

## Discussion

Our meta-analysis provides a comprehensive evaluation of the diagnostic accuracy of [^18^F]FDG PET/CT and [^18^F]FDG PET/MRI in the locoregional assessment of BC. By integrating the most recent evidence, our analysis aims to compare the efficacy of both imaging modalities in the detection of primary BC lesions.

### Primary bladder tumor detection

For the assessment of intravesical BC lesions, our findings indicate that [^18^F]FDG PET/MRI demonstrates superior diagnostic performance compared to [^18^F]FDG PET/CT, with pooled lesion detection rates of 90% and 69%, respectively. This represents a threefold increase in effectiveness based on an indirect comparison of true positive odds ratios. Such an observation is consistent with the known limitations of [^18^F]FDG PET/CT in this domain, primarily due to the high physiological excretion of [^18^F]FDG within the urinary bladder, paired with low-dose CT component [46]. Thus, [^18^F]FDG PET/CT provides suboptimal soft-tissue contrast, further complicating the delineation of intravesical lesions. In contrast, [^18^F]FDG PET/MRI effectively mitigates these limitations through the superior soft-tissue resolution of MRI and the additional functional insights provided by diffusion-weighted imaging [47]. This modality also offers strong concordance with cystoscopic-biopsy findings in primary BC staging via the established Vesical Imaging-Reporting and Data System (VI-RADS) [48]. Additionally, [^18^F]FDG PET/MRI confers the advantage of reduced radiation exposure [49]. While current evidence is compelling, we still need further large-scale studies to validate these findings, particularly given that only three studies to date have investigated [^18^F]FDG PET/MRI as a primary modality for BC primary tumor staging.

### Lymph node staging

With respect to lymph node staging, our study revealed that [^18^F]FDG PET/CT exhibits limited overall sensitivity (50%) but excellent specificity (91%). The suboptimal sensitivity is likely attributable to the inherent spatial resolution constraints of PET, which hinder the detection of subcentimetric lymph nodes [22, 50]. Moreover, paravesical lymph node evaluation remains particularly challenging due to the close proximity of these nodes to the urinary bladder, where intense [^18^F]FDG excretion creates a “sink phenomenon,” obscuring adjacent structures [50]. Additionally, physiological [^18^F]FDG uptake in the distal colon can further confound accurate nodal assessment [51]. Consequently, precise detection of perivesical lymph nodes may necessitate multimodal imaging correlation. Notably, only a single study has assessed the role of [^18^F]FDG PET/MRI in lymph node detection, reporting a sensitivity of 88% [25]. While promising, additional studies are required to establish robust evidence regarding the utility of [^18^F]FDG PET/MRI in nodal staging.

### Technical and protocol considerations

Currently, there is no established consensus on the optimal PET/CT protocol for BC evaluation. While some technical challenges are unavoidable, others may be mitigated through protocol optimization. To enhance sensitivity, several studies have explored various strategies to counteract the sink phenomenon and improve locoregional evaluation [21–23, 37, 42, 43, 45]. These techniques include the administration of diuretics [21, 23, 26, 30, 33, 37, 39, 42, 43], saline boluses [22, 36, 45], urinary bladder catheterization [45], or a combination thereof [21, 37, 43, 45]. Our subgroup meta-analysis demonstrated higher pooled sensitivity in studies that implemented such measures compared to those that did not. Although the difference was not statistically significant, these findings warrant further investigation to optimize PET/CT protocols for improved sensitivity. Sensitivity optimization remains a critical avenue for future research, particularly given the existence of outlier studies that employed these techniques and achieved exceptional sensitivity rates of 100% [21]. Another promising approach involves dual-time-point imaging to monitor lesional [^18^F]FDG uptake dynamics, which has been shown to significantly reduced false-positive findings [21, 37, 43]. Clinicians should consider these strategies to enhance the specificity and accuracy of locoregional evaluation.

### Study limitations

Our meta-analysis has several potential limitations. First, the number of BC patients included in the studies evaluating [^18^F]FDG PET/CT and [^18^F]FDG PET/MRI for primary BC detection remains limited. Further research is necessary to reinforce our findings, particularly in the context of preoperative staging. Second, the predominantly retrospective design of most studies introduces an inherent limitation. Third, our study is limited by reliance on only two online databases, Scopus and PubMed, which may have resulted in missing relevant studies indexed elsewhere. Despite this, our meta-analysis is the first to systematically evaluate primary BC detection using [^18^F]FDG PET/MRI. Moreover, it is the first study to employ subgroup meta-analysis to assess [^18^F]FDG PET/CT in nodal BC staging, delineating sources of heterogeneity and highlighting the impact of PET/CT protocol variations on sensitivity outcomes.

### Future directions

Future research should focus on larger, prospective studies with standardized imaging protocols to better define the roles of [^18^F]FDG PET/CT and [^18^F]FDG PET/MRI in BC evaluation and staging. Optimizing technical protocols and further exploring the potential of PET/MRI for both primary and nodal staging remain critical avenues for advancement.

## Conclusion

Available evidence from a limited number of studies suggests that [^18^F]FDG PET/MRI may offer improved detection rates for primary bladder cancer compared to [^18^F]FDG PET/CT, with preliminary data indicating a detection rate near 90%. However, these findings are based on a small cohort and should be interpreted with caution. While [^18^F]FDG PET/CT maintains high specificity for lymph node staging, its sensitivity requires further optimization. Potential improvements in sensitivity may be achievable through bladder priming, contrast enhancement, and dual-time-point imaging techniques. Further research is warranted to validate these early results and to refine imaging protocols for both modalities.

## Electronic supplementary material

Below is the link to the electronic supplementary material.

Supplementary Material 1

## Data Availability

The data that support the findings of this study are available from the corresponding authors upon reasonable request.
