# Supplementary material for: Diagnostic efficacy of [18F]FDG PET/CT and [18F]FDG PET/MRI in preoperative staging of locoregional urinary bladder cancer: a systematic review and Meta-Analysis
Source: Discov Oncol. 2025 Jul 1;16:1241. doi: 10.1007/s12672-025-03020-1 (PMC12214083; doi:10.1007/s12672-025-03020-1)
Supplement: Supplementary file 1 — Supplementary Material 1 [file 12672_2025_3020_MOESM1_ESM.pdf]

Supplementary Table 1: List of employed MeSH terms.

**Search Concept Group**	**MeSH Term(s) (PubMed/Medline)**
Urothelial/Bladder/Cancer	"Urothelial Carcinoma"[MeSH] OR "Urinary Bladder Neoplasms"[MeSH] OR "Bladder"[MeSH:No]
Cancer/Carcinoma/Neoplasm	"Carcinoma"[MeSH] OR "Neoplasms"[MeSH]
FDG/Fluorodeoxyglucose	"Fluorodeoxyglucose F18"[MeSH]
PET/Positron Emission Tomography	"Positron-Emission Tomography"[MeSH] OR "Tomography, Emission-Computed"[MeSH]
MRI/Magnetic Resonance Imaging	"Magnetic Resonance Imaging"[MeSH]

Supplementary Table 2: Examined metrics of diagnostic test accuracy in each explored domain. FN, False negative; FP, False positive; TN, True negative; TP, True positive.

**Domain**	**Available datasets**	**Number of studies**	**Mode of analysis**	**Pooled Estimate**
Primary tumor detection by [^{18}F]FDG PET/CT	TP, FN	5	Patient-based	Detection rate
Primary tumor detection by [^{18}F]FDG PET/MRI	TP, FN	3	Patient-based	Detection rate
Primary tumor detection: Indirect comparison	TP, FN	8	Patient-based	True-positive odds ratio
Nodal disease staging by [^{18}F]FDG PET/CT	TP, FN, FP, TN	18	Patient-based	Sensitivity, specificity, accuracy

Supplementary Table 3: STARD Checklist

	**Section & Topic**	**No**	**Item**	**Reported on page #**
	**TITLE OR ABSTRACT**			1
		**1**	Identification as a study of diagnostic accuracy using at least one measure of accuracy (such as sensitivity, specificity, predictive values, or AUC)	✓
	**ABSTRACT**			3
		**2**	Structured summary of study design, methods, results, and conclusions (for specific guidance, see STARD for Abstracts)	✓
	**INTRODUCTION**			*4-6*
		**3**	Scientific and clinical background, including the intended use and clinical role of the index test	✓
		**4**	Study objectives and hypotheses	✓
	**METHODS**			*6-9*
	Study design	**5**	Whether data collection was planned before the index test and reference standard were performed (prospective study) or after (retrospective study)	✓
	Participants	**6**	Eligibility criteria	✓
		**7**	On what basis potentially eligible participants were identified (such as symptoms, results from previous tests, inclusion in registry)	✓
		**8**	Where and when potentially eligible participants were identified (setting, location and dates)	✓
		**9**	Whether participants formed a consecutive, random or convenience series	✓
	Test methods	**10a**	Index test, in sufficient detail to allow replication	✓
		**10b**	Reference standard, in sufficient detail to allow replication	✓
		**11**	Definition of and rationale for test positivity cut-offs or result categories of the index test, distinguishing pre-specified from exploratory	✓

		**12a**	Definition of and rationale for test positivity cut-offs or result categories of the reference standard, distinguishing pre-specified from exploratory	✓
		**12b**	Whether clinical information and reference standard results were available to the performers/readers of the index test	✓
		**13a**	Whether clinical information and index test results were available to the assessors of the reference standard	✓
		**13b**	Methods for estimating or comparing measures of diagnostic accuracy	✓
	Analysis	**14**	How indeterminate index test or reference standard results were handled	✓
		**15**	How missing data on the index test and reference standard were handled	✓
		**16**	Any analyses of variability in diagnostic accuracy, distinguishing pre-specified from exploratory	✓
		**17**	Intended sample size and how it was determined	✓
	**RESULTS**			*9-12*
	Participants	**18**	Flow of participants, using a diagram	✓
		**19**	Baseline demographic and clinical characteristics of participants	✓
		**20**	Distribution of severity of disease in those with the target condition	✓
		**21a**	Distribution of alternative diagnoses in those without the target condition	✓
		**21b**	Time interval and any clinical interventions between index test and reference standard	✓
	Test results	**22**	Cross tabulation of the index test results (or their distribution) by the results of the reference standard	✓
		**23**	Estimates of diagnostic accuracy and their precision (such as 95% confidence intervals)	✓
		**24**	Any adverse events from performing the index test or the reference standard	✓
	**DISCUSSION**			*12-14*
		**25**	Study limitations, including sources of potential bias, statistical uncertainty, and generalisability	✓
		**26**	Implications for practice, including the intended use and clinical role of the index test	✓
	**OTHER INFORMATION**			*1, 7*
		**27**	Registration number and name of registry	✓
		**28**	Where the full study protocol can be accessed	✓
		**29**	Sources of funding and other support; role of funders	✓

Supplementary Table 4: Details of indirect Bucher Method

**Parameter**	**Description**
Statistical Test	Bucher's indirect comparison method, which uses variance-weighted comparisons of odds ratios from separate analyses.
Primary Reference	https://doi.org/10.1016/S0895-4356(97)00049-8
Null Hypothesis	The true positive odds ratio difference between [^{18}F]FDG PET/CT and [^{18}F]FDG PET/MRI is not significant

P-value Threshold	Typically set at 0.05 (two-tailed) to determine statistical significance.
Confidence Interval	95% confidence interval (CI) for the odds ratio difference, adjusted for heterogeneity if applicable.
Key Formula	True positive odds ratio difference: ln(OR_PET/CT) – ln(OR_PET/MRI), with variance calculated from study weights.
Interpretation	A confidence interval excluding 1 (for OR) or 0 (for log-OR) indicates statistical significance. Non-overlapping CIs suggest modality superiority.
Indirect Odds Ratio Magnitude Interpretation	- OR ≈ 2: Indicates the odds of the outcome are approximately twofold higher in PET/MRI group compared to PET/CT.<br>- OR ≈ 3: Indicates the odds are approximately threefold higher in PET/MRI group compared to PET/CT.<br>- OR < 1: Indicates the odds are lower in the PET/MRI group compared to the PET/CT. (e.g., OR = 0.5 means the odds are halved).

Supplementary Table 5: Key features of included studies. CF, Clinical follow-up; MIBC, Muscle invasive bladder cancer; NR, Not reported; PA, Pathologic analysis.

Study Name	Year	Country	Sample Size	Age	Male	Female	Study Design	Index Test	Contrast Enhancement	Imaging time	Reference Standard	urothelial cancer%	Prior Neoadjuvant Therapy%	MIBC%
Drieskens 2005	2005	Belgium	55	64	47	8	Retrospective	PET/CT	Without	60	PA or CF	100%	24%	50%
Kibel 2009	2009	USA	42	68	32	11	Prospective	PET/CT	Without	60	PA	100%	0	100%
Swinnen 2010	2010	Belgium	51	66	43	8	Retrospective	PET/CT	With	60	PA	100%	0	71%
Lodde 2010	2010	Canada	70	67	57	13	Prospective	PET/CT	Without	75	PA or CF	73%	0	90%
Jensen 2011	2011	Denmark	18	66	14	4	Retrospective	PET/CT	With	60	PA	100%	NR	67%
Hitier-Berthault 2013	2013	France	52	64	44	8	Prospective	PET/CT	Without	75	PA	94%	0	77%
Goodfellow 2014	2014	UK	93	69	70	23	Retrospective	PET/CT	Without	90	PA	95%	0	90%
Rouanne 2014	2014	France	102	69	80	22	Prospective	PET/CT	Without	60	PA	100%	0	83%
Chakraborty 2014	2014	India	23	60	20	3	Retrospective	PET/CT	With	60	PA	70%	NR	NR
Aljabery 2015	2015	Sweeden	54	68	47	7	Retrospective	PET/CT	With	60	PA	93%	0	74%
Jeong 2015	2015	Korea	61	64	46	15	Prospective	PET/CT	Without	60	PA	100%	0	76%
Soubra 2016	2016	USA	78	68	63	15	Retrospective	PET/CT	With	60	PA	100%	38%	77%
Uttam 2016	2016	India	15	53	14	1	Retrospective	PET/CT	With	60	PA	100%	NR	100%
Pichler 2017	2017	Austria	70	69	53	17	Retrospective	PET/CT	With	60	PA	100%	0	73%
Rosenkrantz 2017	2017	USA	22	66	19	3	Prospective	PET/MRI	With	60	PA	100%	NR	NR
Girard 2019	2019	France	61	73	56	17	Prospective	PET/CT	Without	60	PA	100%	0	100%
Eulitt 2020	2020	USA	18	63	15	3	Prospective	PET/MRI	With	60	PA	100%	72%	100%
Bertolaso 2022	2022	France	85	65	74	9	Retrospective	PET/CT	Without	60	PA	100%	0	100%

Einerhand 2023	2023	Netherlands	237	67	174	63	Retrospective	PET/CT	Without	60	PA	100%	0	68%
Pihl 2023	2023	Sweeden	157	72	116	41	Retrospective	PET/CT	Without	120	PA	NR	0	NR
Longoni 2024	2024	Italy	199	68	168	31	Retrospective	PET/CT	Without	60	PA	100%	32%	82%
Li 2024	2024	China	40	73	32	8	Retrospective	PET/MRI	With	60	PA	100%	0	78%
